# The Hazard Content of Cadmium, Lead, and Other Trace Elements in Some Medicinal Herbs and Their Water Infusions

**DOI:** 10.1155/2017/6971916

**Published:** 2017-10-19

**Authors:** Fuad A. Ababneh

**Affiliations:** ^1^Chemistry Department, College of Science, Al-Hussein Bin Talal University, Ma'an, Jordan; ^2^Chemistry Department, College of Science, Al Imam Mohammad Ibn Saud Islamic University (IMSIU), P.O. Box 90950, Riyadh 11623, Saudi Arabia

## Abstract

54 samples belonging to 23 herbal species (15 individuals and 8 mixtures) were analyzed for their contents of heavy metals in the raw materials and in their water infusions. Trace and toxic elements in these samples were determined by using inductively coupled plasma-atomic emission spectrometry (ICP-OES) following acid digestion. The order of decreasing mean metal concentrations in raw materials (mg/kg) was found to be as follows: Fe (440) > Mn (162) > Zn (45.8) > Cu (12) > Pb (10.4) > Ni (5.4) > Cr (2.9) > Co (0.91) > Cd (0.5). It was found that 21% of the analyzed samples contain both Cd and Pb above their permissible limits. However, the infusions produced from these plants were found to contain fewer amounts of metals than the raw materials. Studied metals had the following mass transfer percentages to the infusion solutions (Fe: 3.5%, Cd: 14%, Cr: 15%, Pb: 20%, Co: 29%, Ni: 31%, Zn: 36%, Cu: 48%, and Mn: 53%). The estimated daily intake calculated for the toxic elements Pb and Cd through the consumption of herbal infusions was far below the tolerable daily intake set by the World Health Organization (WHO).

## 1. Introduction

Medicinal plants and their various products (raw material, mixtures, distillates, extracts, etc.) are well known as herbal medicines; they have been available for many hundreds of years for treatment of human diseases. Nowadays, various species of herbs are widely used as raw materials in the pharmaceutical industries and also extensively consumed as home remedies [1]. Recently, the use of herbal medicines has greatly increased due to mainly the common belief that the herbal remedies are of natural origin and hence inherently safe and without any adverse health effects compared to synthetic medicines. Indeed, this belief is groundless; poisonings associated with the presence of heavy toxic metals in medicinal plants and food were reported regularly [2–4]. Other aspects may encourage the use of herbal remedies such as the availability of herbal medicines without medical prescription, their reasonable low prices, and the fail of synthetic drugs to cure some human diseases [5, 6].

The increasing popularity of herbal products as medications has produced fear about their quality and safety for human consumption. These products may be contaminated with microbial contaminants, chlorinated pesticides, heavy metals, and other chemical toxins [7, 8]. The major sources of metal contamination in herbal products are agricultural activities, mining and smelting industries, rainfall, water and air pollution, soil contamination, and the use of fossil fuels for heating [9]. Metals such as zinc, iron, manganese, and copper are essential elements since they play an important role in biological systems and only become harmful at high concentrations, whereas mercury, lead, and cadmium are nonessential elements as they are toxic, even at low concentration levels [10].

The growing condition of plant, chemical treatments, type of plant species, processing steps, and storage condition are important factors affecting the levels of different metals in herbal preparations [11]. Previous studies showed that some toxic elements were detected in various herbal plants at different concentrations [12–14]. For example, the total arsenic, cadmium, and lead were found in chamomile at concentration ranges of 82–124 *μ*g/kg, 184–256 *μ*g/kg, and 288–1625 *μ*g/kg, respectively [12]. However, in thyme, arsenic and cadmium were found at lower concentrations ranges as 28–67 *μ*g/kg and 143–268 *μ*g/kg, respectively, whereas Pb was found at high elevated concentrations with a range of 6452–8633 *μ*g/kg [12]. The herbal content of the toxic elements only is an indication of the toxicity of these products because of the toxicity affected by the method of exposure. Hence, taking the herbal remedies as an oral drug (consumption as food) will result in the digestion of these products in stomach while taking these products as beverage or as herbal teas means that some of the metals will be extracted to the infusion solution. In this work, the total metal contents and the extracted (fusion) fraction of various herbal preparations will be determined and evaluated for their toxicity.

## 2. Materials and Methods

### 2.1. Reagents and Materials

All solutions were prepared with analytical reagent grade chemicals. Deionized water with specific resistivity of 18.0 M Ω cm^−1^ was used. All glassware used in the present study was previously soaked in 10% (v/v) HNO_3_ solution for 12 h and rinsed with deionized water. Nitric acid (HNO_3_, 69%) and hydrogen peroxide (H_2_O_2_, 30%) were of ultrapure quality (Trace SELECTâ, Fluka). Standard calibration of mixed and individual metal solutions of Fe, Zn, Cu, Cr, Ni, Mn, Co, Pb, and Cd (BDH Spectrosol®, Fluka Chemika®) at concentration of 1000 mg L^−1^ was appropriately diluted and used to calibrate the ICP-OES before metal determination. A certified reference materials (NIST 1570a), spinach leave, obtained from National Institute of Standards and Technology, USA, was used for method validation.

### 2.2. Sampling and Analysis

A total of 54 samples of various commercially available medicinal herbs were randomly purchased from general stores in Irbid city, Jordan. The herbal samples were commercially available in the form of tea bags packaged in boxes with 12–24 pieces of tea bags; each bag contains 1.1–2.7 g of the herbs raw material. The raw herbal material from all tea bags in one box was placed in polyethylene container, mixed, homogenized, and stored at room temperature. Brand codes, contents, and other information are presented in Table 1.

Aliquots (1-2 g) of the herbal raw material were accurately weighed into a precleaned glass beaker and digested with 15 ml of a mixture of freshly prepared solution of (1 : 1) (v/v) HNO_3_ (69%)–H_2_O_2_ (30%). Each beaker was covered with a watch glass and heated on hot plate at 150–180°C; aliquots of nitric acid were added until the solutions were clear. Solutions were contentiously boiled until the volume for each sample reduced to about 5 ml. The solutions were then allowed to cool, filtered (glass wool), and diluted up to 50 ml with acidified (HNO_3_) deionized water and then placed in acid washed 60 ml polyethylene bottles.

The herbal infusions were prepared by adding 200 ml of hot (97°C) Milli-Q water to a weighed portion (2 g) of plant material. The suspension was then stirred with a glass-rod stirrer for 1 minute and steeped for a total of 10 min, as recommended by the manufacturers. The 10-minute infusion time was found to be adequate to prepare a good quality tea beverage [15]. Subsequently, the steeped infusion was filtered and then acidified with HNO_3_ and evaporated to 100 ml.

All digested samples and infusions were analyzed, in triplicate, for metal contents using Inductively Coupled Plasma Optical Emission Spectrometry (ICP-OES) (VISTA-MPX instrument). The simultaneous ICP-OES was equipped with axial vision, a radio frequency (RF) source of 30 MHz, a CCD (Charge Coupled Device), a peristaltic pump, and a glass concentric nebulizer.

### 2.3. Quality Control

In order to validate the instrumental methods and analytical procedures for accuracy, all the samples were taken in triplicate and all measurements were run in triplicate for standards and samples. Analytical blank involving all reagents was run (in triplicate) to check for interferences and cross-contamination of every batch of 15 samples. Certified reference material (NIST 1570a) was also analyzed every batch. Accuracy was determined by comparing the measured concentrations with the certified values and was expressed as a percentage recovery (% recov.). The achieved results were in good agreement with certified values. The results are given in Table 2.

## 3. Results

### 3.1. Mineral Contents of Herbal Raw Materials

The concentrations of Fe, Cu, Zn, Mn, Cr, Co, Ni, Cd, and Pb elements in 54 samples of individuals and mixtures of medicinal herbal plants were determined by using ICP-OES. The mean metals concentrations in raw herbal materials are listed in Table 3. It was observed that all medicinal herbs contain significant amounts of metals with a very wide variability in their concentrations. The order of decreasing mean total concentrations for these metals was Fe > Mn > Zn > Cu > Pb > Ni > Cr > Co > Cd. Similar trend (Fe > Sr > Mn > Zn > Rb > Cu > Ni > Cr > Co > Pb) was reported by another work [16]. It was observed that Fe, Mn, Zn, Cu, and Ni were detected in all of the analyzed herbal samples (100%). Chromium and lead were detected in 87% and 81% of the analyzed samples, respectively. The least detected elements were cadmium and cobalt; they were found in 65% and 54% of the analyzed samples.

### 3.2. Mineral Contents of Herbal Infusion

**Figure 1:**
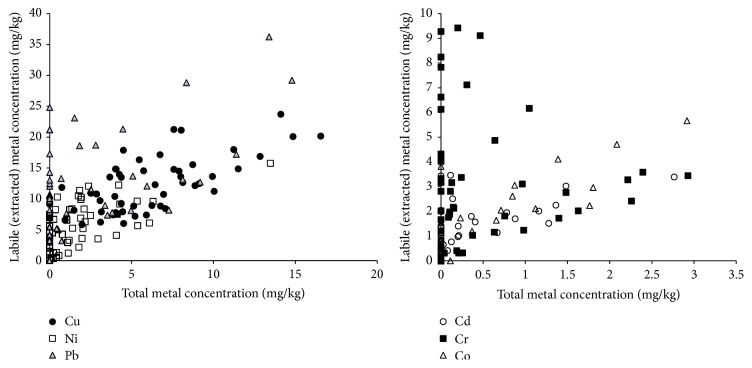
Relationship between Cu, Ni, Pb, Cd, Cr, and Co content of the dried herbal samples and the extracted metal contents of the labile fractions in the infusions.

**Figure 2:**
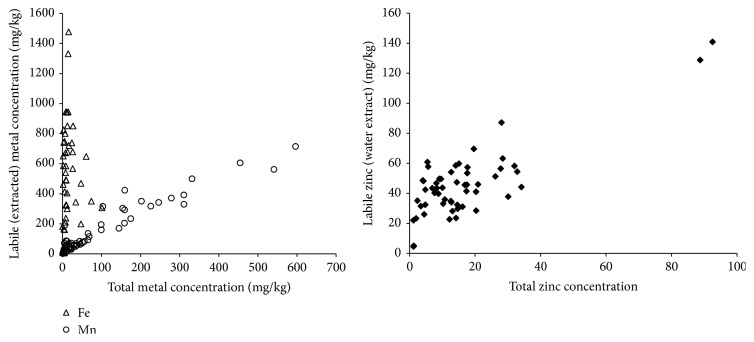
Relationship between Fe, Mn, and Zn contents of the dried herbal samples and the extracted metal contents of the labile fractions in the infusions.

The results for the mean concentrations of studied metals in herbal infusions are presented in Table 4. The mass percentage of metal transported from the raw material to the infusion solution was found to be metal dependent. Iron was found to be the least metal transported from herbs raw material to the infusion solutions; in most cases, less than 10% of total iron in raw material was transported to the infusion solutions with an average of 3.5% for all the analyzed samples. Mn, on the other hand, was found to be the most transported metal to the infusions with an average of 53%. Other metals had the following percentages of mass transfer (Cd: 14%, Cr: 15%, Pb: 20%, Co: 29%, Ni: 31%, Zn: 36%, and Cu: 48%). The correlation between the total metal concentration in raw material and its concentration in the infusion solution (labile fraction) was studied; the results are shown in Figures 1 and 2. Iron and chromium had no significant correlations (*R*-square less than 0.004), while Mn has a strong correlation (*R*-square 0.90). The metals Zn, Co, and Cu had good correlations with *R*-square values of 0.67, 0.64, and 0.5 respectively. Weak correlations were observed for Ni, Pb, and Cd with *R*-square values near 0.34.

The extraction efficiency of metals from the herbal raw material to the water infusion was found to be influenced by the kind of herbal species, while Fe extraction efficiency was below 10% in most cases, it reaches more than 25% for ginger, cumin, and fennel flower. The extraction efficiencies for Zn, Cd, and Pb were high for roselle and exceed 60%. Thyme infusion was found to contain 50% of the initial chromium amount.

## 4. Discussion

The average iron content of the medicinal herbs was in the range of 6.2 mg/kg to 1477 mg/kg with the lowest value in cumin and highest value in peppermint. Samples of chamomile, roots of shirsh zallouh, thyme, and sage had high iron concentrations of 824 mg/kg, 686 mg/kg, 556 mg/kg, and 547 mg/kg, respectively. The mean iron content of all the analyzed samples was 440 mg/kg. In another related work, chamomile and sage had comparable iron contents of 502.7 mg/kg and 297.4 mg/kg, respectively [17]. Similarly, iron concentration range (83.5–405.43 mg/kg) was reported for six herbal teas in another work [18]. Values between 104 mg/kg (white pepper) and 3125 mg/kg (chamomile) had also been reported [19]. However, high iron content (3456 mg/kg) was reported in nettle samples [16].

Manganese mean concentration was 162 mg/kg with a range of 1.8 mg/kg (oliban) to 715 mg/kg (roselle). In addition to roselle; the thyme, cinnamon, ginger, and chamomile samples were found to contain significant amounts of manganese as 324 mg/kg, 251 mg/kg, 250 mg/kg, and 185 mg/kg, respectively. Similar concentration range of manganese (<6 mg/kg to 450.7 mg/kg) was reported for some medicinal plants [20]. However, low manganese content with a range of 1.67 mg/kg to 138.1 mg/kg was reported for ten herbal samples [21]. Indeed, a wide concentration range of 25 mg/kg to 2722 mg/kg was reported for manganese in various plants (herbs, spices, and medicinal plants) [19].

Zinc and cooper were high in roselle (141 mg/kg) and peppermint (23.7 mg/kg), respectively. The mean zinc and copper concentrations in all of the analyzed herbal samples were 45.8 mg/kg and 12.0 mg/kg, respectively. Zinc concentrations varied from 4.6 mg/kg to 141 mg/kg and copper concentrations ranged from 3.37 mg/kg to 23.7 mg/kg. In a related work, similar concentrations ranges of zinc and copper were reported as 3.75–88 mg/kg and 3.32–30.2 mg/kg, respectively [16]. In a recent work [22], copper in powdered herbal medicine was quantified at low concentration range of 0.89 mg/kg to 3.15 mg/kg while zinc had a concentration range of 8.5–38.9 mg/kg. It has also been reported that zinc content of seven herbal plants ranged from 15.4 mg/kg to 73.7 mg/kg and that of copper ranged from 5 mg/kg to 32.7 mg/kg [23].The previous mentioned results were in good agreement with the reported values for both zinc and copper concentrations obtained in this work.

Lead and cadmium are toxic elements for human; they perform no beneficial biological roles and can be very dangerous even at low concentrations. The highest mean concentration of lead in individual herbs was detected in thyme (19.3 mg/kg) followed by oliban (17.7 mg/kg), roselle (17.4 mg/kg), and peppermint (16.2 mg/kg). Among the mixtures of herbs, the Chinese herbal mixture for slimming was found to contain a high mean concentration of lead (28.7 mg/kg). The lowest mean concentration of lead was observed in fennel flower (black seed) and fenugreek where it was below the detection limit (0.06 mg/kg). The mean concentration of lead in all of the analyzed samples was 10.4 mg/kg. Comparable result for the lead concentration range has recently been reported as from below detection limit to 33.8 mg/kg [22]. In related work, thyme was shown to contain 6.45–8.63 mg/kg of lead [12]. On the other hand, low to moderate concentration ranges of lead 0.35 mg/kg–0.82 mg/kg [24], 0.02 mg/kg–3.01 mg/kg [16], 0.26 mg/kg–4.8 mg/kg [17], 0.27 mg/kg–11 mg/kg [25], and 0.04 mg/kg–8.15 mg/kg [26] were reported in various medicinal plants.

The content of cadmium in the analyzed herbs varied between <0.04 mg/kg and 1.27 mg/kg with an average value of 0.5 mg/kg. The highest cadmium content was found in allergic asthma mixture (mix 3) as 1.27 mg/kg and Chinese herbal mixture (mix 8) as 1.25 mg/kg. In the individual herbs, cadmium was detected at high concentrations in roselle (1.01 mg/kg), thyme (0.92 mg/kg), lemon balm (0.69 mg/kg), and sage (0.65 mg/kg). Comparing the results obtained in this research with results found in the literature, the cadmium concentration range is similar to that reported by Onwordil et al. for various types of herbal medicines (0.48–3.08 mg/kg) [22], the results of Subramanian et al. (0.68–2.75 mg/kg) [27], and that of Harris et al. (0.04–4.35 mg/kg) [26]. However, lower mean concentration and concentration ranges than the results of this work were reported. For instance, cadmium concentrations were found in the ranges of below the detection limit to a maximum of 0.44 mg/kg [17], 0.015 mg/kg to 0.268 mg/kg [12], 0.007 mg/kg to 0.27 mg/kg [25], and from below the detection limit to 0.195 mg/kg [28].

The concentration ranges of nickel and chromium are similar; they were measured as from 0.53 mg/kg to 15.7 mg/kg and from <0.03 to 9.43, respectively. The mean nickel and chromium concentrations in all of the analyzed herbal samples were 5.44 mg/kg and 2.9 mg/kg, respectively. In related work, a concentration range of 0.72 mg/kg–13.1 mg/kg with an average of 3.62 mg/kg and a concentration range 0.44 to 8.71 with an average of 2.5 have been reported for nickel and chromium, respectively [16]. Similarly, nickel and chromium concentration ranges were reported as 0.55 mg/kg to 7.31 mg/kg and 0.222 mg/kg–2.4 mg/kg, respectively [29]. The results obtained in this work also agree well with the reported data 0.90–5.4 mg/kg and 0.34 to 1.22 mg/kg for the concentration ranges of Ni and Cr, respectively [17].

Cobalt is the least detected element in the analyzed herbs; it was quantified in 54% of the samples in the range of <0.03 mg/kg to 5.67 mg/kg with mean value of 0.91 mg/kg. High mean concentrations of cobalt were detected in roselle (5.19 mg/kg), sage (1.83 mg/kg), anise (1.6 mg/kg), and peppermint (1.02 mg/kg). In the herbal mixture, high cobalt concentrations were found in allergic asthma mixture (mix 3) (3.92 mg/kg) and coughing mixture (mix 3) (3.59 mg/kg). Comparable results for the cobalt concentration ranges in herbal medicines have been reported as 0.05–2.35 mg/kg [16], 0.08–3.23 mg/kg [29], 0.14–0.48 mg/kg [17], and from below detection limit to 0.87 mg/kg [28].

The extraction efficiencies of various metals from raw material to the water infusions were found to be variable; this was also observed by other related studies. For instance, Queralt et al. [30] showed that the average mass percentage of transported elements to the infusion solution (water at 70°C) are as follows (Mn: 32%, Cu: 30%, Zn: 24%, Pb: 22%, and Fe: 1%); these results are comparable with the results of the present study. In another work, the following sequence of extraction efficiencies was observed (Cd: 53.1%, Pb: 47.5%, Zn: 39.02%, Mn: 19.98%, Cu: 14.4%, and Fe: 10.09%) [26]; the results for Cd, Pb, and Mn were in conflict with the results obtained in the present study. However, in agreement with results of the present study, a recent study showed that the extraction efficiencies for Pb and Cd from raw material of herbs to the infusion solutions were 4.3% and 4.1%, respectively [31]. In another related work, the extraction efficiencies of various metals from ten herbs were reported as Zn: 35%, Cu: 33%, Mn: 24%, and Fe: 6% [32]. In a recent work, the leaching of Ni and Mn to the infusion solutions was reported as the highest, while Fe was the least [33]; the mentioned results were in good agreement with the present study results.

Because of the toxic properties of lead and cadmium, it is important to evaluate the safety of the herbal preparation. In this work the two toxic elements lead and cadmium were quantified in the raw materials of herbal remedies with average values close to or slightly above their permissible limits of 10 mg/kg and 0.3 mg/kg, respectively. It was found that 40% of the analyzed samples contain more than 10 mg/kg of lead and 60% of the analyzed samples contain more than 0.3 mg/kg of cadmium, while 30% of the samples contain the two elements above their permissible limits. Indeed, the determination of total concentration of the toxic elements in the herbal raw materials gives indication of the toxicity of the herbal preparation, but this step is not sufficient in assessing the safety of these products because the consumers take these remedies as recommended by the producers as their infusion in hot water. In fact, all infusions were found to contain fewer amounts of metals than the raw materials, and so it is important to evaluate the infusions for their safety. By assuming the consumption of 200 ml infusion prepared from 2-scahets (2 g) two times a day, as recommended by the manufacturer, and by using the mean value of the % extraction efficiency for Pb and Cd, the estimated daily intake was calculated as 8.32 *μ*g/day and 0.28 *μ*g/day for Pb and Cd, respectively. These values were far below the tolerable daily intake of Pb (0.3 mg/day) and Cd (1.5 ug/day). The amounts of essential trace elements were very low in the infusions, and the estimated daily intake was calculated as 0.06 mg/day, 0.07 mg/day, 3.36 *μ*g/day, and 23 *μ*g/day for Fe, Zn, Cr, and Cu, respectively. It is clearly calculated that there is no health risk associated with the consumption of these herbal products with respect to their metal contents. However, the content of Pb and Cd must be monitored frequently to avoid the accumulation of these toxic elements.

## 5. Conclusions

Quantification of nine metal elements (Fe, Mn, Zn, Cu, Cr, Co, Ni, Pb, and Cd) in the raw materials and water infusions of selected herbal remedies and comparison with the values in the literature revealed that the heavy metal contents of these commercially available products vary and are affected by the plant species. In the raw materials, Fe had the highest mean content (440 mg/kg) and Cd had the lowest mean content (0.5 mg/kg). Mn and Cu demonstrated relatively high extraction efficiencies to the infusions while Fe had the lowest extraction efficiency. Some raw herbal materials had lead and cadmium contents above the permissible limits. However, the water infusions of these medicinal preparations contain lower levels of lead and cadmium and consumption of the tested beverages poses no risk to human health.

## Figures and Tables

**Table 1:** Summary of the medicinal herbal samples, their common and scientific names, and sources and their recommended medicinal uses.

Sample code	Brand name	Number of samples	Common name	*Scientific name*	Product of (source)	Medicinal uses (claimed on the manufacturers' packaging)
H1	Teebah	4	Sage	*Salvia officinalis *	Jordan	Digestive problems, depression, memory loss, diarrhea, bloating, and overproduction of perspiration
H2	Ibn Sina	2	Cumin	*Cuminum cyminum*	Syria	Antiflatulence antispasmodic and diuretic menstruation
H3	Al-Bothoor	2	Oliban	*Boswellia carterii*	Egypt	Chest coughs, nausea, indigestion, and hypertension
H4	Al-Khodary	4	Chamomile	*Matricaria recutita*	Jordan	Anti-inflammatory, sleep aid, treatment for fevers, colds, and stomach ailments
H5	Al-Nakhah	3	Peppermint	*Mentha piperita*	Jordan	Irritable bowel syndrome and colonic spasms, functional dyspepsia, and enhancement of gastric emptying
H6	Al-Rawabi	2	Cinnamon	*Cinnamomum cassia*	Jordan	Control of blood sugar levels, powerful anti-bacterial agent, and natural disinfectant
H7	Al-Attar	2	Anise	*Pimpinella anisum*	Syria	Remove phlegm and catarrh in the bronchial passageways, relieve asthma symptoms, indigestion and stomach pain, and mouth freshener
H8	Bint -Alreef	2	fennel flower (black seed)	*Nigella sativa*	Jordan	Healing for all diseases
H9	Al-Nakhah	2	Ginger	*Zingiber officinale*	Jordan	Inflammation of the colon, asthma symptoms, pain during menstruation, high blood pressure chronic inflammation, and relief of nausea
H10	Al-Rawabi	3	Thyme	*Thymus vulgaris*	Jordan	Kidney problems, muscle pain, chronic fatigue, parasites, anxiety, and headache
H11	Al-Bothoor	2	Roselle	*Hibiscus sabdariffa*	Egypt	High blood pressure, coughs, and colds
H12	Ibn Sina	2	Lemon Balm	*Melissa officinalis*	Syria	Nervous agitation, urinary spasms, functional gastrointestinal complaints, sleep disorders, and menstrual cramps
H13	Bint -Alreef	2	Fenugreek	*Trigonella foenum-graecum*	Jordan	Laxative, digestive aid, remedy for coughs and bronchitis, and increases in breast milk production
H14	Teebah	2	Cardamom	*Elettaria cardamomum*	Jordan	Digestion problems, common cold, cough, and bronchitis
H15	Al-Nakhah	3	Roots of shirsh zallouh	*Ferula hermonis*	Jordan	Enhance sexual performance
M1	Al-Attar	3	Herbal tea (zhourat) mixture		Syria	Antimicrobial, antiviral, and inflammation of gastric mucosa
M2	Al-Attar	2	Herbal mixture	—	Syria	Sedative of renal colic
M3	Al-Attar	2	Herbal mixture	—	Syria	Sedative allergic asthma
M4	Al-Attar	2	Herbal mixture	—	Syria	Coughing
M5	Al-Attar	2	Herbal mixture	—	Syria	Cold
M6	Al-Attar	2	Herbal mixture	—	Syria	Inflation and gases
M7	Al-Attar	2	Herbal mixture	—	Syria	Laxative
M8	Ibn Sina	2	Mixed Chinese herbs	—	Syria	Slimming

**Table 2:** Concentrations of selected elements in the NIST1570a certified reference material.

Metal	Certified value mg/kg	Measured value mg/kg	Recovery %
Mn	75.9	72	94.9
Zn	82	84	102.4
Ni	2.14	2.0	93.5
Co	0.39	0.36	92.3
Cu	12.2	11.3	92.6
Cd	2.89	2.59	89.6

**Table 3:** The average contents of heavy metals (mg/kg) in the tested raw medicinal herbal samples.

Sample code	Zn	Fe	Cu	Co	Cr	Mn	Ni	Cd	Pb
H1	54.2 ± 8.6	574 ± 260	8.6 ± 3.4	1.8 ± 1.3	2.1 ± 0.9	99 ± 136	5.9 ± 3.7	0.7 ± 0.4	9.2 ± 7.7
H2	51.8 ± 11.4	6.60 ± 0.55	13.5 ± 1.8	<0.03	2.7 ± 0.7	28.7 ± 9.1	6.34 ± 2.7	0.2 ± 0.04	5.5 ± 3.2
H3	4.9 ± 0.4	49.5 ± 3.9	7.9 ± 0.7	<0.03	3.7 ± 0.6	2.1 ± 0.3	1.9 ± 0.9	0.5 ± 0.1	17.7 ± 4.8
H4	49.8 ± 3.3	824 ± 344	12.6 ± 1.3	<0.03	1.52 ± 1.3	185 ± 209	3.05 ± 2.8	0.17 ± 0.34	8.01 ± 5.4
H5	39.6 ± 12	1121 ± 308	16.9 ± 5.9	1.02 ± 1.1	3.10 ± 0.6	148 ± 41	4.45 ± 3.7	0.63 ± 0.58	16.2 ± 3.1
H6	24.7 ± 1.9	171 ± 14.7	7.12 ± 5.3	0.04 ± 0.04	<0.04	251 ± 92	0.67 ± 0.93	0.25 ± 0.36	7.90 ± 0.36
H7	72.3 ± 21.1	86.2 ± 111	13.9 ± 4.5	1.65 ± 0.56	2.1 ± 2.7	57.7 ± 0.2	3.98 ± 4.9	0.38 ± 0.15	5.33 ± 3.3
H8	59.4 ± 2.2	7.4 ± 0.6	16.3 ± 2.2	0.77 ± 0.13	1.8 ± 0.2	20.5 ± 6.2	3.91 ± 2.4	<0.01	<0.04
H9	26.3 ± 4.9	136 ± 89	6.70 ± 1	<0.03	3.2 ± 4.1	250 ± 112	2.4 ± 1.6	0.62 ± 0.06	11.0 ± 1.4
H10	46.5 ± 11.8	556 ± 139	17. 3 ± 2.4	0.29 ± 0.5	0.71 ± 0.09	324 ± 252	2.47 ± 1.7	0.92 ± 0.5	19.3 ± 10.6
H11	135 ± 8.5	328 ± 30.5	14.7 ± 3.1	5.19 ± 0.67	5.52 ± 0.9	660 ± 77	12.7 ± 4.3	1.01 ± 0.9	17.4 ± 10.4
H12	38.9 ± 15.1	437 ± 33.6	9.79 ± 1.3	0.60 ± 0.8	1.88 ± 2.2	69.3 ± 2.7	6.01 ± 3.3	0.69 ± 0.9	6.56 ± 9.2
H13	36.4 ± 5.5	24.8 ± 4.2	9.13 ± 1.7	<0.03	<0.04	26.5 ± 15.1	0.53 ± 0.8	<0.01	<0.01
H14	36.7 ± 6.1	311 ± 16.1	17.5 ± 3.7	0.07 ± 0.4	0.21 ± 0.29	382 ± 14	4.27 ± 1.39	0.40 ± 0.16	8.26 ± 4.61
H15	35.3 ± 13.2	686 ± 108	7.77 ± 1.17	<0.03	6.74 ± 2.47	39.7 ± 20	8.40 ± 1.8	0.66 ± 0.26	8.14 ± 4.33
M1	45.6 ± 5.7	519 ± 321	14.5 ± 6.2	0.18 ± 0.3	3.75 ± 3.8	353 ± 65	7.25 ± 6.17	0.44 ± 0.5	5.74 ± 9.9
M2	52.9 ± 7.6	693 ± 64	6.89 ± 0.9	<0.03	2.40 ± 0.6	69.7 ± 17.6	8.38 ± 2.1	<0.01	18.20 ± 6.9
M3	56.6 ± 18.4	800 ± 72	15.8 ± 3.1	3.92 ± 0.14	2.00 ± 0.17	85.2 ± 10.8	11.2 ± 1.3	1.27 ± 0.63	18.6 ± 8.2
M4	40.9 ± 7.0	570 ± 114	10.5 ± 2.3	3.59 ± 0.74	2.99 ± 0.26	76.7 ± 10	10.9 ± 0.62	<0.01	6.1 ± 4.0
M5	41.0 ± 4.5	282 ± 63	8.5 ± 1.1	1.87 ± 0.33	8.67 ± 0.61	182 ± 31	6.2 ± 1.5	0.38 ± 0.1	9.2 ± 5.3
M6	37.5 ± 8.4	13.1 ± 5.3	17.9 ± 4.7	0.78 ± 1.1	8.27 ± 1.6	56.4 ± 9.6	7.7 ± 1.0	0.15 ± 0.05	18.6 ± 26.0
M7	26.1 ± 3.5	62.4 ± 18.3	7.3 ± 1.1	<0.03	2.94 ± 0.24	24.9 ± 7.4	4.6 ± 2.3	0.76 ± 0.34	4.24 ± 1.3
M8	37.3 ± 5.9	783 ± 59	12.3 ± 3.6	0.34 ± 0.48	1.48 ± 0.34	276 ± 59	4.2 ± 0.8	1.25 ± 0.36	28.7 ± 10.5

Statistical values of all 54 samples									
Highest	141	1477	23.7	5.67	9.43	715	15.7	1.25	28.7
Lowest	4.63	6.23	3.37	nd	nd	1.82	0.53	nd	nd
Mean	45.8	440	12.0	0.91	2.90	162	5.44	0.5	10.4
Median	43.7	409	11.5	0	2.29	79.9	5.40	0.4	9.25
St. dev.	23.1	357	4.62	1.44	2.60	172	3.82	0.45	8.44

**Table 4:** Average contents of heavy metals (mg/kg) in the raw samples and water infusions of the medicinal herbs.

Element	Zn	Fe	Cu	Co	Cr	Mn	Ni	Cd	Pb
Dried herbs (mg/kg)	45.8 ± 5.1	440 ± 77	12 ± 1.0	0.91 ± 0.3	2.9 ± 0.6	162 ± 38	5.4 ± 0.8	0.5 ± 0.1	10.4 ± 1.8
Infusions (mg/kg)	16.5 ± 3.7	15.6 ± 4.3	5.8 ± 0.8	0.26 ± 0.1	0.4 ± 0.1	86 ± 9	1.7 ± 0.5	0.07 ± 0.1	2.1 ± 0.8
Extraction efficiency (%)	36 ± 4	3.5 ± 4	48 ± 5	29 ± 5	15 ± 7	53 ± 6	31 ± 7	14 ± 6	20 ± 5
